# Improving rates of pneumococcal vaccination on discharge from a tertiary center medical teaching unit: A prospective intervention

**DOI:** 10.1186/1471-2458-5-110

**Published:** 2005-10-14

**Authors:** Chandra M Thomas, Andrea Loewen, Carla Coffin, Norman RC Campbell

**Affiliations:** 1 Department of Medicine, University of Calgary, Calgary, Canada

## Abstract

**Background:**

Pneumococcal disease causes significant morbidity and mortality in at-risk individuals, and is complicated by emerging antibiotic resistance. An effective, safe and cost-effective vaccine is available, but despite this many patients who would benefit from pneumococcal vaccination remain unvaccinated. The purpose of this study was to determine the rates of missed opportunities to provide pneumococcal vaccination to patients being discharged from a tertiary center medical teaching unit and to determine if a nurse coordinator-based intervention would increase rates of pneumococcal vaccination prior to discharge home.

**Methods:**

We conducted a prospective, controlled study in the setting of a Medical Teaching Unit at a tertiary care centre to assess the impact of a nurse coordinator based intervention on the rates of vaccination of eligible patients on discharge home. The rates of vaccination during an eight-week usual-care period (February 20 to April 16, 2002) and an eight-week intervention period (April 22 to June 16, 2002) were compared.

**Results:**

Prior to the intervention none of thirty-eight eligible patients were vaccinated prior to discharge home from the Medical Teaching Unit. After the intervention 27 (54%) of fifty eligible patients were vaccinated prior to discharge.

**Conclusion:**

There are significant missed opportunities to provide pneumococcal vaccination to inpatients who are discharged home from a medical unit. Using a patient care coordinator we were able to significantly improve the rates of vaccination on discharge.

## Background

Invasive infection by *streptococcus pneumoniae *is an important cause of morbidity and mortality, particularly in individuals older than 65 years of age, and those with chronic health conditions. The incidence of invasive pneumococcal disease almost triples in those over 65. Despite appropriate antibiotic therapy and supportive care there is a high case fatality rate in pneumococcal bacteremia of 15–20% in adults overall with highest rates in those over 65 and those with chronic medical conditions. The case fatality rate has been reported as 12.1% for individuals aged 16 to 64 with indications for vaccination compared to 5.4% to individuals of the same age with no indications [1].

Previously there was uniform susceptibility to penicillins. The National Centre for Streptococcus in Edmonton, Alberta now has reported 10.2% of isolates have diminished susceptibility to penicillin [2]. Macrolide resistance among invasive *S. pneumoniae *isolates also dramatically increasing [3].

Given the high morbidity and mortality of pneumococcal disease, and increasing antibiotic resistance, the focus is shifting to primary prevention. The 23-valent pneumococcal vaccine is between 50–80% effective against invasive pneumococcal disease in older and high-risk individuals [4]. The vaccine is also cost-effective in high-risk individuals [5,6].

The rates of vaccination against pneumococcus are well below targets and studies have shown significant missed opportunities for vaccination of at-risk individuals [7]. The Health Canada target vaccination rate is 80% of patients who meet the Canadian Immunization Guidelines criteria for pneumococcal vaccination [8]. However, only 42% of those over 65 and 15% of those eligible persons younger than 65 have received pneumococcal vaccination [8]. Studies have shown that 36% to 70% of patients admitted to hospital with invasive pneumococcal infection had been inpatients in the five years prior to admission [9,10]. Brull et al demonstrated that pneumococcal vaccine is the most commonly overlooked preventive health intervention among medial patients who are discharged from a tertiary care hospital [11]. Another study showed that a computerized reminder system in a teaching hospital resulted in a pneumococcal vaccination rate of 35.8% of eligible patients compared to 0.8% in the control group [12]. Many hospitals do not have specialized computer systems such as that described in this study, therefore the generalizability of the study is limited.

Given the evidence that there was room for improvement in the delivery of pneumococcal vaccination to at-risk individuals discharged from hospital, we embarked on a study to determine if a nurse-coordinator-based intervention would increase the rate of pneumococcal vaccination of eligible patients discharged home from an inpatient medical teaching unit.

## Methods

Our study was conducted on the medical teaching unit (MTU) at Foothills Medical Centre, a tertiary referral centre in Calgary, Canada with over 700 inpatient beds. The MTU had an average patient census of 20 to 30 with a median length of stay of 7 days. Primary medical care was provided by 3 to 4 clinical clerks and 4 to 5 junior residents under the supervision of 2 senior residents and an attending physician. The team also had a dedicated clinical pharmacist and a care coordinator. The care coordinator is a registered nurse who routinely meets with patients on the day of admission to describe the structure of the MTU, liaises with the patient's outpatient primary care and specialist physicians and coordinates appropriate resources and follow-up for the patient on discharge from hospital.

Patients were considered to be eligible for our study if they met Canadian Immunization Guidelines criteria to receive pneumococcal vaccination, these criteria are described in Appendix 1 [8]. Patients who died, were transferred to other services or had been vaccinated prior to admission were excluded. Patients transferred to other services included those transferred to critical care services and surgical services for surgical intervention. Medically stable patients for which a protracted hospitalization was anticipated were transferred to a non-teaching service prior to discharge home or to long term care facilities.

The first admission was used for patients admitted multiple times to the medical teaching unit. Patients admitted during the usual-care period were eligible to be included in the intervention period if they continued to meet eligibility criteria.

Previous vaccination status was determined by asking the patient and by reviewing the Calgary Public Health vaccination database, which includes information on all pneumococcal vaccinations given in the Calgary Health Region since 1997.

At the outset of the study a multidisciplinary team comprised of internal medicine residents, attending internists, an MTU Care Coordinator, a pharmacist, a nurse manager and a representative from public health was assembled. The team noted that there was no process in place to ensure that eligible patients would be vaccinated on discharge. They embarked on the task of developing a flowchart that would represent a rational process that, if followed, would result in vaccination of patients who were discharged home from the MTU.

**Figure 1:**
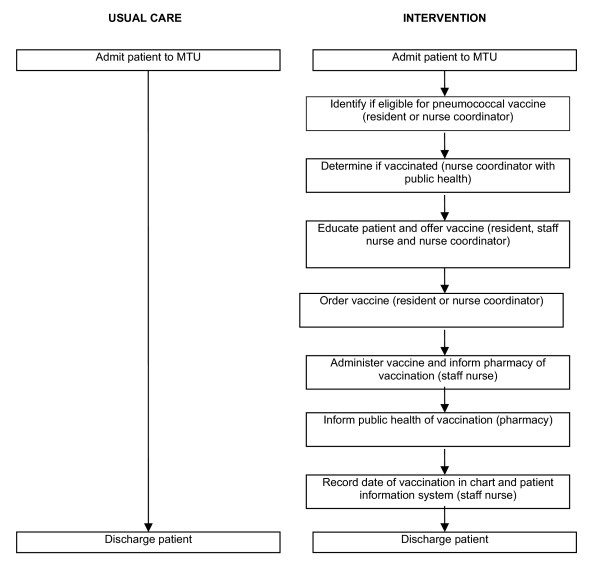
The study algorithm for vaccinating patients discharged during the usual-care and intervention periods.

The final process is diagrammed in Figure 1.

The final study consisted of a pre and post intervention where an assessment of pneumococcal vaccine provision on discharge home took place during a usual care period and then an intervention period where the process described in figure 1 and outlined below in the 'Intervention Period' was implemented.

### Usual-care period

The usual care period consisted of an eight-week period where the medical students and residents on the MTU were provided with a standardized form outlining eligibility for pneumococcal vaccination. These team members were responsible for assessment of patient eligibility, determining vaccination status and, if appropriate, ordering a dose of vaccine. However, no advice on how best to carry out these tasks was provided. The MTU Care Coordinator determined vaccine eligibility and gathered information on previous vaccination status and whether or not a vaccine dose was ordered at the time a patient was discharged from hospital. This information was not provided to the medical students or residents. The usual care period extended from February 20 to April 16, 2002.

### Intervention period

The intervention period involved rolling out the process described above and outlined in Figure 1. The first step in the process included a formalized method of identification of eligible patients. A standardized eligibility form was developed and a consistent individual, the MTU care coordinator, was given the responsibility of ensuring that the form was completed. The completed eligibility form was faxed to the public health office where the database was examined and the vaccination status of the patients admitted to the MTU was reported to the MTU care coordinator. Patients who were eligible to receive the pneumococcal vaccine but had not been previously vaccinated were provided with educational materials on the vaccine by the MTU care coordinator who would, in concert with the floor nurses, housestaff and the attending internist, answer any patient questions on the vaccine. The patient was then offered the vaccine and, if they agreed, it was ordered by the housestaff on the team. The MTU care coordinator oversaw this process and would ensure that the responsible housestaff ordered the vaccine. Nursing staff administered the vaccine as ordered and completed a vaccination form that was sent to the pharmacy. The pharmacy would forward these forms on to public health so that the vaccination database could be updated. All of the individuals and procedures described above were present prior to the intervention but were not previously linked together by a responsible individual – the care coordinator.

A nursing inservice was held on April 17, 2002 to inform staff of the initiative, the background issues and to review vaccine administration and charting. At this time the vaccination rates from the initial 8 week assessment were posted for nursing staff and residents. On April 22, 2002 a computerized message appeared on terminals throughout the Foothills Medical Centre reminding staff to vaccinate eligible patients against pneumococcus on discharge. From April 22 to June 16, 2002 the process described above was implemented.

### Statistical analysis

The overall characteristics of patients admitted to the MTU and those for the usual-care and the intervention period were described using means or medians for continuous variables and proportions for categorical variables. Differences between the groups were assessed using Student's t-test. The vaccination rates during the study period were compared using the Chi-square test. All analyses were carried out using Stata Version 5 [13].

## Results

**Figure 2:**
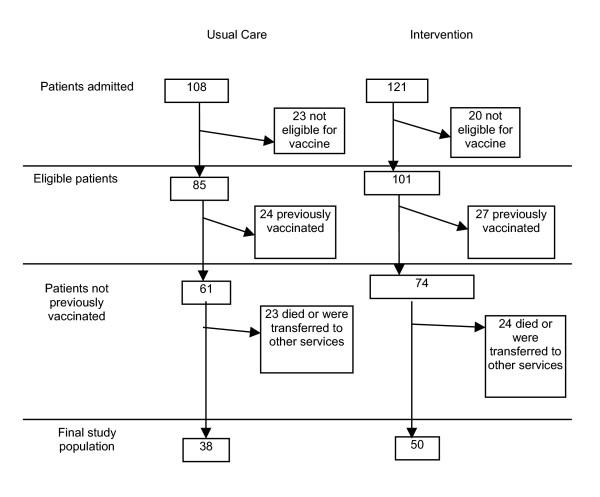
The flow of patients in the intervention and usual care periods of the study.

The final study population consisted of 38 individuals in the usual-care group and 50 individuals in the intervention group (Figure 2). There were 229 individuals admitted to the MTU during the two eight-week study periods (108 in the usual-care period and 121 during the intervention period). Eight individuals had multiple admissions. Of the patients admitted to the MTU 80.8% (n = 186) were eligible to receive pneumococcal vaccine. Ninety-one (48.9%) of patients were eligible because they were aged greater than 65 years. For patients less than 65 years of age the most common reason for eligibility was liver disease 45.3% (n = 43). Fifty-one (27.4%) of the individuals who met criteria for vaccination had been vaccinated prior to admission, 15.7% of the patients less than 65 years of age and 39.6% of the greater than 65 years age group, and were, therefore excluded from the final study population. Sixty-five percent of eligible patients who were not previously vaccinated were discharged home leaving a final study population of 88 patients.

The characteristics of the final study population by intervention group are described in Table 1. The study population was similar prior to and during the intervention with no significant difference between the two groups with respect to age, length of stay or sex. The primary study outcome was vaccination rate on discharge home. Fifty-four percent (n = 27) of patients in the intervention group were vaccinated prior to discharge home compared to none of the patients in the usual-care group. The vaccination rate for individuals greater than 65 years of age tended to be higher than the younger group (64.7% versus 48.5%). However, this difference was not statistically significant (p = 0.276). Individuals vaccinated on discharge also had a longer median length of stay (5.5 versus 10 days) but the interquartile ranges for these two values overlap (i.e. the difference was not statistically significant).

**Table 1:** Characteristics of study population

Characteristic	Usual-care (n = 38)	Intervention (n = 50)
Age (mean with 95% CI)	56.1 (50.9–61.2)	54.7 (49.7–59.6)
<65 years of age % (n)	63.2% (24)	66% (33)
Reason for vaccine eligibility if age < 65 years*		
Splenectomy	1.5% (1)	3.0% (1)
Cardiovascular disease	15.4% (10)	6.1% (2)
Pulmonary disease	13.9% (9)	6.1% (2)
Liver disease (includes alcoholism)	30.8% (20)	51.5% (17)
Renal disease	4.6% (3)	9.1% (3)
Diabetes mellitus	16.9% (11)	24.2% (8)
Immunosuppressed	6.2% (4)	24.2% (8)
Length of stay (median with IQ range)	7 (3–14) days	7 (4–11) days
Female (%)	52.6% (20)	38.0% (19)

*percentages add up to more than 100 because individuals are present in more than one category

**Figure 3:**
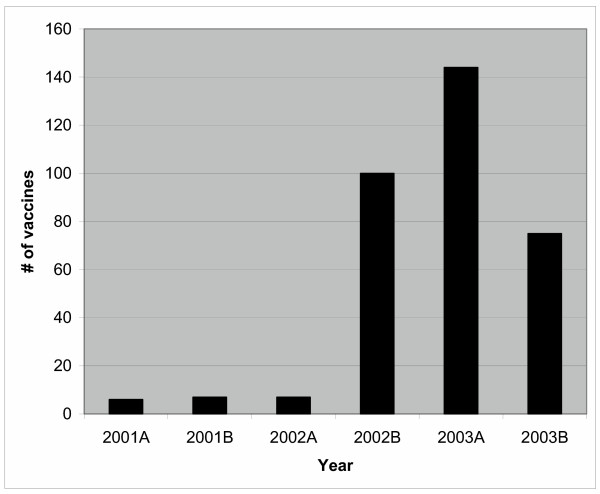
Pneumococcal vaccination use in Foothills Hospital*. * The number of vaccinations administered in the Foothills Hospital prior to and following the study. The time periods were chosen to correspond with the study periods in 2002 – usual-care period from Feb. 20 to April 16 and the intervention period from April 22 to June 16. The bar graph shows an increase in administration of pneumococcal vaccinations after the intervention and indicating there were significant effects outside of the unit where the intervention occurred and that the effect was sustained after the study was terminated.

To determine if the study had a broader impact on prescribing of pneumococcal vaccines in the Foothills Hospital, we examined ordering of pneumococcal vaccine outside of the MTU setting. In addition to the 27 patients vaccinated on the MTU, 73 patients were vaccinated on other services. Vaccine use was assessed in the same time periods as the study in the year prior to and after the study. There was an increase from 13 vaccinations in 2001 to 219 in 2003 while the Foothills hospital bed capacity increased by only 36 beds (April 2001 761 beds, April 2002 766 beds and April 2003 797 beds). This information is presented in Figure 3.

## Discussion

We demonstrated that there are significant missed opportunities to provide pneumococcal vaccination to eligible patients discharged home from a medical teaching unit service. Further, we demonstrated that by a collaborative, multidisciplinary effort and with the involvement of a nurse coordinator, we were able to assess 100% of patients for eligibility and reach a vaccination rate of 54% [14,15]. Additionally, one year after the intervention the use of pneumococcal vaccine remains increased compared to prior to this study. The successful intervention involved developing a sustainable process for vaccinating patients on discharge by reorganizing existing resources.

The vaccination rate in this study was superior to that which is currently achieved on an outpatient basis. Furthermore, it was also superior to the improvement that was shown in a recent study assessing the impact of a highly specialized computerized reminder system that found a rate of 35.8% after their intervention suggesting that a multimodality intervention is superior to ensure vaccination on discharge [12]. Other interventions that utilized computer-generated admissions list and pre-printed order forms for the vaccine and a dedicated nurse vaccine manager did not manage to achieve rates of vaccination as high as that which was reached in our study. We think the superior results achieved in our study stem from the systematic process developed using integral team members to ensure vaccination on discharge and there being a single responsible individual being accountable.

Based on the known efficacy of the vaccine, and its cost effectiveness in high risk populations, we would anticipate that this intervention will diminish invasive pneumococcal disease, hospitalizations and health care costs in the future.

One limitation in this study was vaccine supply. The hospital pharmacy had anticipated an increase in vaccine utilization. However, the degree of increased vaccine ordering far exceeded that which was anticipated and there was a one week time period when patients were unable to receive vaccination prior to discharge due to lack of availability. A further limitation was that occasionally patients were discharged and left hospital prior to receiving an ordered vaccination. We were unable to quantify the number of theses cases. The continuity of a nurse coordinator in the setting of a MTU where housestaff are constantly rotating was pivotal, and this approach will be most applicable on services where there is a nurse care coordinator. A strength of our study was the presence of a population-based database of vaccination. This database allowed the intervention to taget unvaccinated patients. Without the database 27% of patients entering the hospital could have been revaccinated.

A meta-analysis of interventions that increase adult immunization supports organizational change as the most effective means of improving preventive care measures, surpassing financial incentives, patient reminders, patient education and feedback [16]. We found also that organizational change, with the involvement of a nurse coordinator to do specific prevention activities was effective. With the flux of staff on the MTU the long-term sustainability of this intervention remains to be proven.

## Conclusion

There are significant missed opportunities to provide pneumococcal vaccination to hospital inpatients who are at high risk from pneumococcal pneumonia. The use of a patient care coordinator and minor system changes significantly improved the rates of vaccination on discharge.

## List of abbreviations

MTU: Medical teaching Unit

## Appendix

Guidelines for use of pneumococcal vaccination [7].

• All persons ≥65 years of age

• All persons >2 years with the following: asplenia, splenic dysfunction or sickle cell disease

• All persons >2 years with the following chronic conditions: chronic cardiorespiratory disease (except asthma), cirrhosis, alcoholism, chronic renal disease, nephritic syndrome, diabetes mellitus, chronic CSF leak, HIV infection and other conditions associated with immunosuppression (Hodgkins disease, lymphoma, multiple myeloma, induced immunosuppression for organ transplantation

## Competing interests

The author(s) declare that they have no competing interests.

## Authors' contributions

CT, AL and CC collectively assisted in designing, conducting the research and revising the manucsript. CT conducted the statistical analysis and drafted the manuscript. NC supervised the research project and assisted in all aspects of the trial, and revising the manuscript.

## Pre-publication history

The pre-publication history for this paper can be accessed here:


